# Poor results of drilling in early stages of juxta-articular osteonecrosis in 12 joints affected by Gaucher disease

**DOI:** 10.3109/17453670902930032

**Published:** 2009-04-01

**Authors:** Ehud Lebel, Mici Phillips, Deborah Elstein, Ari Zimran, Menachem Itzchaki

**Affiliations:** ^1^Department of Orthopaedic SurgeryJerusalemIsrael; ^2^Department of Orthopaedic Gaucher Clinic, Shaare Zedek Medical CenterJerusalemIsrael

## Abstract

**Background and purpose** Gaucher disease is heterogeneous. One of the most devastating complications is bone involvement, ranging from mild osteopenia to osteonecrosis, but no markers have been discovered to predict onset and/or progression. We describe our experience in a large referral center using drilling for juxta-articular osteonecrosis in young patients with Gaucher disease.

**Patients and methods** We retrospectively reviewed medical data from all patients who were recommended to undergo drilling for osteonecrosis of juxta-articular bone of the femoral head, the humeral head, or upper tibia for acute osteonecrosis at a pre-collapse stage.

**Results** 11 patients (mean age 34 years) underwent drilling of 12 joints with juxta-articular osteonecrosis; 3 (mean age 51 years) refused intervention. 9 joints that were drilled showed advancing joint degeneration within 0.5 to 4 years. 3 joints have undergone replacement. Of the 3 joints that did not undergo drilling, 2 have undergone replacement and 1 has collapsed with osteoarthritis.

**Interpretation** We found equally poor outcome with and without drilling. Effective intervention can only be achieved by improving our understanding of bone physiology and pathophysiology in Gaucher disease.

Gaucher disease is the most common lysosomal storage disease, and occurs in approximately 1 in 50,000 live births. It is more common in Ashkenazi Jews where it occurs in approximately 1 in 850 live births (Beutler and Grabowski 2001). There is accumulation of lipids due to deficient β-glucocerebrosidase, but neither enzyme activity nor other genetic or biochemical biomarkers can predict onset or severity of disease progression (Beutler and Grabowski 2001). One of the most devastating consequences of the disease is bone involvement, which affects most patients (Elstein et al. 1997). The underlying pathology of bone in Gaucher disease is unclear, but it is postulated to be secondary to bone marrow infiltration by lipid-laden macrophages, causing vascular occlusion or a local inflammatory reaction (Cox 2001). There are recognized risk factors for bone disease in Gaucher disease (Rodrigue et al. 1999) such as splenectomy (especially in childhood) and the presence of alleles that produce little or no enzyme. Bone involvement ranges in severity from discrete radiographic findings such as the Erlenmeyer flask deformity of the distal femur and the “herringbone” pattern of the humerus diaphysis, to osteopenia and osteonecrosis (Itzchaki et al. 2004).

Enzyme replacement therapy (ERT) (Genzyme Corp., Cambridge, MA) improves the visceral and hematological features of the disease (Barton et al. 1991) as well as sense of well-being (Giraldo et al. 2005). Even so, the effect of ERT on bone remains controversial since there is not necessarily a correlation between radiological improvement (Poll et al. 2002) and clinical lack of deterioration (Elstein et al. 1996). ERT may eliminate bone crises if treatment is begun early (Charrow et al. 2007), but ERT does not appear to reverse existing osteonecrosis. To date, there are no definitive theories to explain the inadequate response of affected bone to ERT.

Invasive interventions have been recommended for the pre-collapse stages of femoral osteonecrosis in otherwise healthy patients (Mont et al. 2006), to prolong time to replacement (McGory et al. 2007). Core decompression was used by Ficat (1983) with good results (79% success rate in patients with disease of stages I–II). When used in sickle cell anemia and compared to physical therapy, however, no additional benefit was noted (Neumayr et al. 2006). Yet, it is possible that the bone marrow may be impacted directly by decompression in sickle cell disease if attempted early in the progression to collapse (Hernigou et al. 2006).

The cause of osteonecrosis in Gaucher disease may be in the marrow; thus, drilling of affected bones in Gaucher disease seems tenable. We report our experience using drilling for joint osteonecrosis in patients with Gaucher disease.

## Patients and methods

We retrospectively reviewed all patients recommended to undergo small-diameter drilling for osteonecrosis of juxta-articular bone of the femoral head, the humeral head, or upper tibia for acute osteonecrosis in a pre-collapse stage, ARCO stages 1–2 (Gardeniers 1993). Excluded from the current report were patients sustaining nonarticular osteonecrosis (which is not uncommon in Gaucher disease), and those in whom late results of osteonecrosis were diagnosed (collapse was already evident in the hip, knee, or shoulder).

Of 618 patients diagnosed as having Gaucher disease (by enzymatic testing and genetic analysis), 11 patients had drilling of 12 joints. 3 additional patients refused intervention. None of these patients were lost to follow-up.

Diagnosis of osteonecrosis was based on acute joint pain and verified by MR imaging. Sphericity of the joint head was assessed by radiographs and CT. Staging of the lesion was based on the ARCO system for femoral osteonecrosis with modifications for other bones, but using the same evaluators as for the hip.

**Figure 1.**
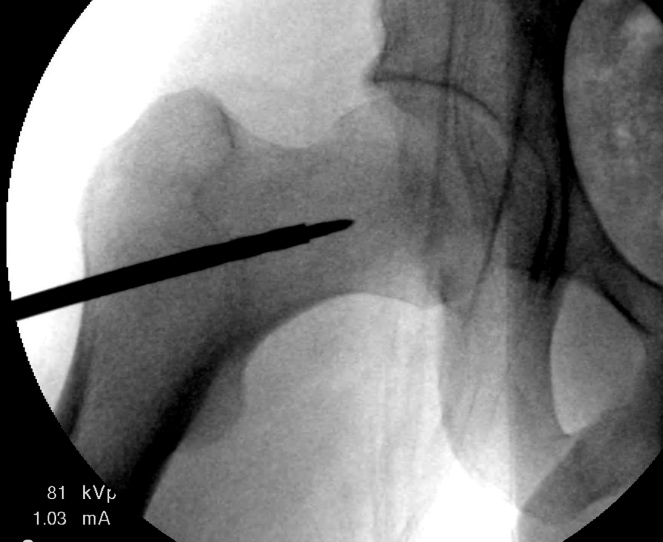
Typical view of Gaucher bony milieu (taken during drilling).

The size of lesions is not stated in this report since radiological findings within the bone marrow are evident in all cases but delineation of borders of osteonecrosis and Gaucher-related lesions are difficult to define. Invariably (and universally), the medullary lesions in Gaucher bone are accompanied by abnormal matrix composition (Figure 1) that often raise conflicting radiological signals and complicate the reading of acute or chronic involvement. The presence of artifacts on MRI further confuses attempts at delineation of the borders of the affected area.

11 patients underwent drilling (Table 1) and 3 did not accept drilling (Table 2). The patients were followed for 2–16 years.

**Table 1.** Demographic data of all patients who have undergone drilling

Patient	Sex	Age at event	Splenectomy	Genotype	Enzyme replacement therapy at event	Affected bone	ARCO stage at diagnosis **^a^**
1	M	26	No	1226/1226	Yes	Femoral head	1
2	M	21	No	1226/84GG	No	Femoral head	2
3	F	33	No	1226/84GG	Yes	Proximal tibia	2
4	M	47	Yes	1226/1226	No	Femoral head	2
5	F	32	No	1226/1448	Yes	Humeral head	1
6	M	41	No	1226/1226	No	Humeral heads	2, 2
7	M	42	Yes	1226/IVS	No	Femoral head	2
8	M	36	No	1226/?	No	Femoral head	1
9	M	15	Yes	1226/84GG	No	Femoral head	1
10	M	26	No	1226/1226	No	Femoral head	1
11	M	31	No	1226/1226	No	Femoral head	1

**^a^** for explanation of ARCO staging, see text.

**Table 2.** Demographic data of patients who did not agree to intervention

Patient	Sex	Age	Splenectomy	Genotype	Enzyme replacement therapy	Affected bone	ARCO stage at diagnosis	Evolution (time/status)
1	F	33	No	1226/1226	Yes	Femoral head	1	1 year / hip replacement
2	M	51	No	1226/84GG	Yes	Femoral head	1	1 year / hipreplacement
3	M	69	No	1226/1226	Yes	Proximal tibia	1	3 years / stage 3 (osteoarthritis of knee **^a^**)

**^a^** Kellgren class.

Drilling was done percutaneously with a 5-mm drill bit under general or spinal anesthesia and using fluoroscopic guidance. After cortical perforation, the drill was advanced into the osteonecrosis (according to MRI or radiographic findings) while 4–6 medullary tracts were drilled into the necrotic region. In 3 patients (numbers 1–3), autologous bone marrow was aspirated from the ipsilateral iliac crest and injected into the drilled lesion. The lateral cortex perforation was left open in all cases. In 1 patient (number 8), contrast medium was injected into the drilled tunnel to improve visualization. Drains were not used. Postoperatively, all patients were encouraged to resume full range of joint motion and were advised to use crutches to prevent weight bearing on the treated limb (in the femur and tibia cases) for 6 weeks. Perioperative antibiotic was used for no more than 24 h. No complications occurred.

## Results (Table 3)

**Figure 2.**
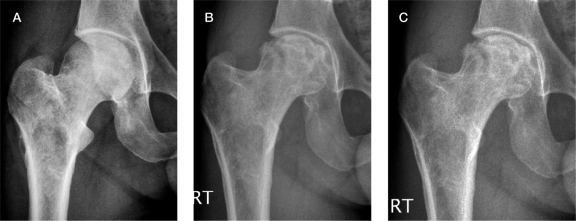
Chronological sequence in patient 10, who did not develop articular collapse and joint derangement. At presentation (A), at 1.5 years (B), and at 2 years (C).

2 of the 11 drilled patients did not develop articular collapse and joint derangement; these patients are currently free of pain and have functional joint motion (1 shoulder with 16 years of follow-up and 1 hip with 4 years of follow-up). These 2 patients (numbers 5 and 10, respectively) had osteonecrotic lesions farther from the joint surface than the others (Figure 2).

**Table 3.** Outcome in patients undergoing drilling

Patient	Affected bone	ARCO stage at drilling	Time from diagnosis and drilling	Years to ARCO stage 4
1	Femoral head	1	1 week	1.5
2	Femoral head	2	10 months	2
3	Proximal tibia	2	4 months	2
4	Femoral head	2	3 months	2
5	Humeral head	1	14 months	No progression
6	Humeral heads	2, 2	8 months	4, 4
7	Femoral head	2	1 month	1
8	Femoral head	1	11 months	1
9	Femoral head	1	18 months	3
10	Femoral head	1	2 months	No progression
11	Femoral head	1	2 weeks	0.5

All other patients (9 joints) showed advancing joint degeneration within 0.5–4 years. Of these 8 patients, 5 underwent hip joint replacement by prosthetic implant. 1 patient (number 2) underwent hip-adductor tenotomy to improve hip range of motion and currently shows joint ankylosis but is unwilling to consider any further intervention. The remaining patients (numbers 1 and 6) are currently not in need of surgical intervention but have evidence of a degenerative joint.

All 3 patients who elected for no intervention progressed to articular surface collapse. The 2 patients with femoral head involvement underwent total hip replacement within 2 years of osteonecrosis. The patient with tibial plateau depression uses a knee brace and ambulates freely.

## Discussion

Osteonecrosis of the femoral head, the humeral head, or the tibial knee surface is a devastating consequence of Gaucher disease. Core decompression or drilling can change the course of osteonecrosis in its early stages in populations without Gaucher Disease (Steinberg et al. 2001), but this type of intervention for early stages of osteonecrosis in Gaucher disease has never been studied.

For the current cohort, drilling was recommended immediately, i.e. when articular cartilage damage was not yet evident (ARCO stages 0–2). Thus, intervention should have prevented subchondral collapse and subsequent degeneration of the joint. Surprisingly, local complications such as fracture, infection, or substantial bleeding were not encountered, negating a long-standing belief that patients with Gaucher disease are prone to infection and bleeding. However, this highlights the poor outcome of intervention itself in the current cohort: only 2 of 12 joints have not progressed to osteoarthritis. Moreover, these 2 were the only joints where the lesions were quite distant from the joint, and possibly for this reason there was no collapse.

Drilling of necrotic bone is believed to accelerate osteoclast recruitment, substitution of dead bone, release of large amounts of bone morphogenic proteins and other factors from bone, platelets, endothelium, and monocytes (Seyler et al. 2007), thus accelerating bone healing. Our findings of lack of efficacy of intervention by drilling raise two hypothetical explanations. Either the process as diagnosed by imaging techniques is different from what we diagnose as osteonecrosis of other etiologies (Mont et al. 2007) or some factor(s) in the medullary milieu in Gaucher disease prevent(s) healing.

Elevated serum levels of cytokines of the TNF superfamily have been noted in Gaucher disease (Allen et al. 1997, Barack et al. 1999). Such high levels might negate the osteoblast induction effect of bone drilling. Gaucher cells (lipid-engorged macrophages) might also have toxic effects in this milieu (although fracture healing is normal in patients with Gaucher disease). Bone collapse was evident even in a non-weight bearing bone such as the head of humerus, demonstrating that limiting ambulation will not necessarily prevent collapse.

Drilling of necrotic bone might actually accelerate osteoclastic activity in a non-favorable marrow homeostasis. The osteoclast-osteoblast balance of bone resorption coupled with new bone formation may not be preserved in Gaucher disease (leading to osteopenia and a high prevalence of trabecular bone fractures). Thus, adding osteoclast inhibitors such as bisphosphonates might help to prevent aggressive bone resorption without laying new trabecular bone that will support subchondral bone. Only 1 patient (number 1) was already receiving alendronate because of inclusion in a clinical trial (Wenstrup et al. 2004) before osteonecrosis occurred, and yet collapse occurred at least as quickly as in the other cases.

We can only hypothesize that our findings are more comparable to those seen in sickle cell anemia than in other bone pathologies, which indicate a lack of responsiveness by the marrow to clear the dead bone and/or to induce new trabecular bone formation under conditions of Gaucher-induced osteonecrosis. Whether this also indicates that the bony necrosis in Gaucher disease is inherently unique—or that a secondary (epigenetic) set of factors such as those induced by inflammation and inflammatory cytokines is detrimental to bone—cannot be determined from our clinical observations.
